# Alpha1-antitrypsin impacts innate host–pathogen interactions with *Candida albicans* by stimulating fungal filamentation

**DOI:** 10.1080/21505594.2024.2333367

**Published:** 2024-03-22

**Authors:** Martin Jaeger, Axel Dietschmann, Sophie Austermeier, Sude Dinçer, Pauline Porschitz, Larsen Vornholz, Ralph J.A. Maas, Evelien G.G. Sprenkeler, Jürgen Ruland, Stefan Wirtz, Tania Azam, Leo A.B. Joosten, Bernhard Hube, Mihai G. Netea, Charles A. Dinarello, Mark S. Gresnigt

**Affiliations:** a Department of Medicine, University of Colorado Denver, Aurora, USA; b Department of Internal Medicine, Radboud University Medical Center and Radboud Center for Infectious diseases (RCI), Nijmegen, the Netherlands; c Junior Research Group Adaptive Pathogenicity Strategies, Leibniz Institute for Natural Product Research and Infection Biology – Hans Knöll Institute, Jena, Germany; d Department of Microbial Pathogenicity Mechanisms, Leibniz Institute for Natural Product Research and Infection Biology – Hans Knöll Institute, Jena, Germany; e Institute of Clinical Chemistry and Pathobiochemistry, School of Medicine and Health, Center for Translational Cancer Research (TranslaTUM), Munich, Germany; f Department of Laboratory Medicine, Laboratory of Hematology, Radboud University Medical Center, Nijmegen, The Netherlands; g Medizinische Klinik 1, Universitätsklinikum Erlangen, Friedrich-Alexander-Universität Erlangen-Nürnberg, Erlangen, Germany; h Institute of Microbiology, Friedrich-Schiller-University, Jena, Germany; i German Cancer Consortium (DKTK), partner site Munich, Germany; j German Center for Infection Research (DZIF), partner site Munich, Germany

**Keywords:** Immune escape, immune evasion, host–pathogen interactions, fungal adaptation, filamentous growth, cell wall remodelling

## Abstract

Our immune system possesses sophisticated mechanisms to cope with invading microorganisms, while pathogens evolve strategies to deal with threats imposed by host immunity. Human plasma protein α1-antitrypsin (AAT) exhibits pleiotropic immune-modulating properties by both preventing immunopathology and improving antimicrobial host defence. Genetic associations suggested a role for AAT in candidemia, the most frequent fungal blood stream infection in intensive care units, yet little is known about how AAT influences interactions between *Candida albicans* and the immune system. Here, we show that AAT differentially impacts fungal killing by innate phagocytes. We observed that AAT induces fungal transcriptional reprogramming, associated with cell wall remodelling and downregulation of filamentation repressors. At low concentrations, the cell-wall remodelling induced by AAT increased immunogenic β-glucan exposure and consequently improved fungal clearance by monocytes. Contrastingly, higher AAT concentrations led to excessive *C. albicans* filamentation and thus promoted fungal immune escape from monocytes and macrophages. This underscores that fungal adaptations to the host protein AAT can differentially define the outcome of encounters with innate immune cells, either contributing to improved immune recognition or fungal immune escape.

## Introduction

Infections caused by the yeast *Candida albicans* are among the primary opportunistic fungal nosocomial infections [1,2]. Systemic candidiasis, characterized by dissemination of fungi to vital organs, results in a mortality of approximately 200,000 deaths annually worldwide [3].

Protective host defence against *C. albicans* is mainly mediated by efficient fungal clearance by neutrophils, monocytes and macrophages, whereas the fungus employs a variety of pathogenicity mechanisms to counteract these processes [4]. In case of *C. albicans* this includes the switch to filamentous growth, facilitating fungal invasion of host tissues and complicating successful phagocytosis by innate immune cells [5,6]. Integration of host environmental signals can induce fungal adaptations with a strong impact on fungus–host interactions driving immune evasion and escape [7].

α1-antitrypsin (AAT) is a potent immunomodulatory acute phase protein, whose plasma concentration increases during infection [8]. AAT inhibits serine proteases relevant to the immune system such as elastase and proteinase-3 [9,10], while also exhibiting immunomodulatory effects independent from its protease inhibitory activity [11]. Clinically, AAT is used for supplementation in deficient patients [12], and also is explored as an immunomodulatory treatment for graft versus host disease [13,14].

Previous studies suggest a potential role for this protein during *C. albicans* infection: genetic variation in the *SERPINA1* gene encoding AAT, has been associated with susceptibility to candidemia [15] and *SERPINA1* expression is significantly upregulated in peripheral blood mononuclear cells (PBMCs) in response to *C. albicans* [15,16].

While the pleiotropic immunomodulatory effects of AAT have been well characterized, its role in host–pathogen interactions with *C. albicans* remains unresolved. In the present study, we investigated how AAT impacts host–pathogen interactions by assessing its effect on the function of various innate immune cells (neutrophils, monocytes, and macrophages) after stimulation with *C. albicans*.

## Methods

### Study approval

All experiments with human blood samples were performed and conducted in accordance with good clinical practices, the Declaration of Helsinki, the approval of the Arnhem-Nijmegen Ethical Committee (no. 2010/104) and the Jena Institutional Ethics Committee (Ethik-Kommission des Universitätsklinikums Jena, Permission No 2207–01/08). Blood from volunteers was taken after written informed consent was obtained.

### PBMC, neutrophil, and CD14 monocyte isolation and macrophage differentiation

Human PBMCs were isolated from buffy coats or freshly drawn venous blood. Isolation of PBMCs was performed by density gradient centrifugation over Histopaque-1077 (Sigma Aldrich) in a 50 mL sterile tube. Neutrophils were isolated from the erythrocyte/granulocyte fraction using hypotonic lysis in 155 mM NH_4_Cl, 10 mM KHCO_3_. Afterwards, neutrophils were washed twice in PBS and resuspended in RPMI-1640 media with 2 mM L-glutamine (Thermo Fisher Scientific). CD14^+^ monocytes were isolated from the PBMC fraction using magnetic beads and automated cell sorting (autoMACs; MiltenyiBiotec). Neutrophils and monocytes were seeded at 1 × 10^5^ cells per well, respectively, in 96-well plates and used for experiments immediately. To differentiate monocytes into human monocyte-derived macrophages (hMDMs), 1.7 × 10^7^ cells were seeded into 175 cm^2^ cell culture flasks in RPMI-1640 media with 2 mM L-glutamine (Thermo Fisher Scientific) containing 10% heat-inactivated foetal bovine serum (FBS; Bio&SELL) and 50 ng/mL recombinant human macrophage-colony-stimulating factor (M-CSF; Immunotools, Friesoythe, Germany) for 7 days with a full medium exchange on day 5. Human macrophages were detached from cell culture flasks by 20 min exposure to 10 mM EDTA and cell scraping on day 7 and reseeded at 4 × 10^4^ per well in 96-well plates and rested over night before experiments.

### Differentiation of mouse bone marrow-derived macrophages

Collection of bone-marrow was approved by the local ethics committees of the Technical University of Munich and Erlangen University clinic. Mouse bone marrow of WT, *Card9-/-* [17] and *Gsdmd-/-* [18] was cultured for 7 days in the presence of 50 ng/mL recombinant mouse M-CSF (Peprotech, Cranbury, NJ) with a full medium exchange on day 5. BMDMs were detached and reseeded as described for human macrophages.

### *C. albicans* strains and culture

Wild-type *C. albicans* strains SC5314 [19], and BWP17 Clp30 [20], the non-filamentous mutants *efg1Δ/Δ/cphΔ/Δ* [21] and *hgc1Δ/Δ* [22], the candidalysin deficient mutant *ece1Δ/Δ* [23], the cell wall mutant *och1Δ/Δ* [24] and an *pECE1*-GFP strain [23] were used. *C. albicans* strains were grown from glycerol stocks on YPD agar. Single colonies were picked and grown in YPD at 30°C overnight while shaking at 180 rpm. For the other experiments, yeasts were washed in PBS, and density was adjusted to the desired inoculum level.

### Fungal killing assays

Human neutrophils (10^5^/well), CD14^+^ monocytes (10^5^/well), MDMs (4 × 10^4^/well) or mouse BMDMs (4 × 10^4^/well) were seeded in 96-well plates. Cells were allowed to engulf and kill live *C. albicans* cells (MOI 1 for neutrophils, monocytes, and hMDMs, and MOI 2.5 for BMDMs) for 3 h or 24 h at 37°C and 5% CO_2,_ respectively. Killing assays were performed in RPMI-1640 media with 2 mM L-glutamine (Thermo Fisher Scientific) in the presence or absence of human alpha-1 antitrypsin 1–1000 µg/mL (AAT, Zemaira, CSL Behring, lyophilized reconstituted in water at 50 mg/mL). Following incubation, the well contents were plated on YPD agar plates and incubated overnight at 37°C. After incubation, *C. albicans* colony forming units (CFUs) were quantified using an automated colony counter (ProtoCOL 3; Synbiosis).

### Quantification of fungal escape by macrophage lysis

For analysis of macrophage lysis kinetics, we adapted a published method [25]. Briefly, 4 × 10^4^ cells/well (hMDMs) were seeded in 96-well plates. Macrophages were then infected with *C. albicans* (MOI 1) in the presence of 1–1000 µg/mL AAT, and propidium iodide (PI; 3.33 µg/ml; Sigma Aldrich) was added to stain macrophages that lost membrane integrity. Alternatively, 4 × 10^4^ mouse BMDMs were infected with *C. albicans* MOI 2.5 in presence of 0.5 µM SytoxGreen (Invitrogen) instead of PI. Infected macrophages were incubated at 37°C and 5% CO_2_ and imaged in a Zeiss Cell Discoverer 7 microscope with an integrated AxioCam 506 controlled using Zeiss Zen software. Four independent fields/well were imaged at the optimal interval possible (15–60 min) intervals at 10× (NA 0.35) magnification for a maximal period of 24 h using the bright field channel and DsRed or SytoxGreen filters. The red and green channel images were processed using the FIJI software [26]. After conversion to binary images, the PI/SytoxGreen-positive cells were enumerated using the Particle Analyzer tool and macro batch analysis. The average total number of macrophages per field of view was determined by lysing macrophages using 0.1% Triton-X100 in PBS and counting PI or SytoxGreen positive macrophages. By normalizing to these values, a percentage of cell death over-time could be calculated.

### AAT binding to fungal surface

*C. albicans* was seeded in 8-well ibidi µ-slides at 3 × 10^4^ CFU/well and grown for 2 h in RPMI. Afterwards, AAT was added to a concentration of 1 mg/mL for another hour under the same conditions. Cells were carefully washed with PBS and fixed 20 min in 4% Histofix (Roth). Cells were washed and blocked in PBS containing 1% BSA for 30 min RT. The primary antibody (unlabelled polyclonal rabbit IgG anti-AAT #711079, Invitrogen) was incubated diluted 1:200 overnight at 4 °C. Cells were washed with PBS, stained for 2 h at RT with 20 µg/mL goat-anti-rabbit-AlexaFluor647 (Invitrogen) and washed again. After the last washing step, the fixed *C. albicans* cells were covered with PBS and imaged on a Cell discoverer microscope (Zeiss) at 40× magnification.

For flow cytometry detection, SC5314 yeasts were cultured shaking at 37 °C in presence of 1 mg/mL AAT for 1 h. Cells were washed with FACS buffer (PBS, 2% FCS) and stained with anti-AAT (unlabelled polyclonal rabbit IgG, Invitrogen) at 4 °C overnight. The next day, cells were washed with FACS buffer and stained with goat-anti-rabbit-AlexaFluor647 (Invitrogen) at 4°C for 20 min. Cells were filtered through a 70 µm mesh before acquisition on a FACSVerse Cell Analyzer (BD Biosciences, Franklin Lakes, NJ). Analysis was performed using FlowJo V10.

### *C. albicans* transcriptional profiling

*C. albicans* was grown in 6-well plates at 1 × 10^6^ CFU/well in RPMI-1640 medium or medium containing 100 µg/mL AAT. After 3 h of incubation at 37°C and 5% CO_2_, the supernatant was removed and replaced with RNeasy Lysis (RLT) buffer (Qiagen), containing 1% β-mercaptoethanol (Roth). The cells were detached using a cell scraper and snap-frozen in liquid nitrogen. Samples were thawed on ice and centrifuged for 10 min (20,000 × g, 4°C). The supernatant was discarded, and fungal RNA was isolated from the pellet, using the freezing-thawing method, as described previously [27]. Fungal RNA concentrations were quantified using a NanoDrop 1000 Spectrophotometer (Thermo Fisher Scientific), and RNA quality was assessed with Agilent 2100 Bioanalyzer (Agilent Technologies). RNA was subsequently converted into Cy5-labelled cRNA (Cy5 CTP; GE Healthcare, United Kingdom) using a QuickAmp labelling kit (Agilent). Samples were co-hybridized with a common Cy3-labelled reference (RNA from mid-log-phase-grown *C. albicans* SC5314[28]). *C. albicans*-specific microarrays (ClinEuroDiag) were applied as previously described [29]. Two micrograms of the Cy3- and Cy5-labelled cRNA were hybridized to the *C. albicans* arrays overnight at 42°C in DIG Easy Hyb solution (Roche). The slides were washed at RT using gene expression wash buffer 1 and 2 (Agilent), acetonitrile, and stabilization and drying solution (Agilent) according to the manufacturer’s protocol. Hybridized slides were scanned with an Axon 4000B scanner at a 10 µm resolution. Data were extracted by GenePix 4.1 software (Axon). Data processing was performed in R using packages provided by Bioconductor 2.26 [30]. The microarray data were pre-processed using the limma package [31]. “Printtiplowess” normalization was used on each array separately to correct for spatial effects or cross-hybridization. Array spots corresponding to the same gene were summarized using the duplicated correlation function of limma. Normalization of the arrays was performed using between-array quantile normalization. Gene expression was compared between each experimental condition and the common reference using limma. Subsequently, expression values were compared between non-treated and AAT-treated *C. albicans* using gene-spring (Agilent). Genes with a Benjamini-Hochberg corrected *p*-value <0.05 (FDR) and a log_2_ Fold change of > 0.8 or < -0.8 between experimental conditions were considered differentially regulated. Unsupervised clustering analysis was performed in R using the pheatmap package [32], and distances were defined using Euclidean distance. Principle component analysis was performed using the prcomp function in R. Heatmaps of selected gene sets were plotted using the pheatmap package in R [32]. GO term enrichment of differentially expressed genes was analysed using the GO-Term Finder on the *Candida* genome database [33], which uses a hypergeometric distribution with Multiple Hypothesis Correction (Bonferroni Correction) to calculate *p*-values.

### *C. albicans* filamentation

*C. albicans* was seeded in 96-well plates at 1 × 10^4^ cells/well in the presence or absence of 1–1000 µg/mL AAT in RPMI-1640 medium after which microscopic imaging of the *C. albicans* cells was performed. In selected experiments, *C. albicans* was only grown in PBS in the presence or absence of 1–1000 µg/mL AAT. For imaging of *C. albicans* hyphae formation, the plate was incubated at 37°C and 5% CO_2_ in a Zeiss Cell Discoverer 7. Microscopic pictures were taken at a 10× (filamentation measurements) or 40× (representative images of WT, *efg1Δ/Δ/cphΔ/Δ* and *hgc1Δ/Δ* filamentation) magnification. For the images obtained at 3 h, hyphae length was quantified using the line tool in FIJI software [26]. The percentage of germination was quantified in a constant defined area per image and classified based on their specific morphology (yeast or hyphae). The images were analysed in a blinded manner.

### Measurement of *p-ECE1*-GFP expression

From 19 h filamentation assays in PBS the percentage of GFP expressing fungi was quantified. GFP-channel images were converted to 32 bit in FIJI software [26], and using threshold and the particle analyser tool the number of positive cells was quantified. The number of non-GFP expressing fungi were quantified from a merged image of the GFP and brightfield channels.

To measure fluorescence intensity of the *p-ECE1*-GFP strain, GFP-channel images were converted to 32 bit in FIJI software [26], using the threshold setting Over/Under the background was separated from the fluorescently stained fungi, using the ROI manager and the wand tool, stained fungi were randomly selected and intensity was measured. The images were analysed in a blinded manner.

### Stimulation of cytokine release

Monocytes and macrophages were stimulated in 96-well flat bottom plates with either live *C. albicans* yeast in the presence of AAT or *C. albicans* that had been fixed with 4% Histofix (Roth, Karlsruhe, Germany) after being exposed to AAT. For the latter the 1 × 10^5^ *C. albicans* cells per well were grown for 3 h in presence or absence of AAT, carefully washed in PBS and then fixed, after which they were washed again 4 times, carefully. After 3 h or 24 h at 37°C and 5% CO_2_ cells were centrifuged at 300 × g, and supernatants were collected and stored at -20°C until cytokine measurements were performed by ELISA according to the instructions of R&D Systems ELISA DuoSets for human (or mouse) IL-1β and TNF.

### Immunofluorescence staining of cell wall molecules

*C. albicans* was seeded in 8-well ibidi µ-slides at 3 × 10^4^ CFU/well and grown for 3 h in RPMI or RPMI containing AAT (1 or 10 µg/mL). Subsequently, the fungi were fixed for 15 min in Histofix (Roth). Cells were stained following an adapted protocol of the previously published method for mannan, β-glucan, and chitin staining [34]. In brief, cells were washed and blocked in PBS containing 1% BSA, after which β-1,3-glucan was stained by incubating for 30 min with mouse anti-β-1,3-glucan (10 µg/mL; Biosupplies, Bundoora, Australia) in PBS with 1% BSA. Subsequently, the unbound antibody was removed by washing in PBS containing 1% BSA. Bound antibodies directed to β-glucan were secondarily stained for 30 min using goat anti-mouse Alexa Fluor 488 (5 µg/mL; Invivogen) in PBS with 1% BSA. Mannan was stained by incubating fungi for 30 min with Concanavalin-A conjugated with Alexa Fluor 647 (25 µg/mL; Invitrogen) in PBS. Chitin was stained by incubating fungi for 30 min with calcofluor white (10 µg/mL, CFW, fluorescent brightener; Sigma) in 100 mM TRIS-HCl pH 9.5. For triple staining, mannan and chitin were stained following β-glucan staining using a staining mixture of ConA-647 and CFW in 100 mM TRIS-HCl pH 9.5. Stained cells were mounted using ProLong™ Gold Antifade Mountant (ThermoFisher Scientific). Microscopy pictures were taken with a Zeiss observer microscope and attached AxioCamMRm3 at 10× and 40× magnification.

To measure fluorescence intensity, colour images were converted to 32 bit using the FIJI software [26]. By using the threshold setting Over/Under the background was separated from the fluorescently stained fungi, using the ROI manager and the wand tool, stained fungi were randomly selected and intensities were measured. Two independent individuals separately assessed the images in a blinded fashion.

### Quantification of fungal uptake

3 h co-cultures of human MDMs (4 × 10^4^ cells/well in a 96-well plate) and SC5314 (MOI 1) were fixed 20 min in 4% Histofix, washed and blocked with 1% BSA in PBS for 15 min at 37°C. To mark extracellular fungal structures, samples were stained with Concanavalin-A conjugated with Alexa Fluor 647 (25 µg/mL) in the blocking buffer for 1 h at RT. Cells were washed and permeabilized with 0.5% Triton-X in PBS and stained with 10 µg/mL Calcofluor white for 45 min at RT to mark extra- and intracellular fungal elements. After washing, the images were acquired on a Cell Discoverer 7 microscope (Zeiss). All fungi with a ConA^+^ mother cell were considered extracellular, all fungi with CFW^+^/ConA^−^ mother cells intracellular. These events were quantified manually with Fiji’s cell counter plugin in a blinded manner. A ratio was calculated via dividing intracellular by extracellular events.

CD14^+^ monocytes (10^5^/well) were allowed to engulf FITC-labelled *C. albicans* for 2 h in the presence or absence of 10 µg/mL AAT. Subsequently, the fluorescence signal of extracellular non-phagocytosed *C. albicans* cells was quenched using trypan blue, as previously described [35]. The monocytes that phagocytosed one or more quantified based on their positivity for the FITC signal were quantified on a Beckman Coulter CytoFlex.

### Fungal killing in the presence of β-1,3-glucan masking

CD14^+^ monocytes (10^5^/well) were seeded in 96-well plates and were allowed to engulf and kill live *C. albicans* cells (MOI 1) for 24 h at 37°C and 5% CO_2_. The *C. albicans* was either prepared with a control IgG or mouse anti-β-1,3-glucan (10 µg/mL; Biosupplies, Bundoora, Australia). Killing assays were performed in RPMI-1640 media with 2 mM L-glutamine (Thermo Fisher Scientific) in the presence or absence of human alpha-1 antitrypsin (0, 1, or 10 µg/mL; Zemira, CSL Behring). Following incubation, the well contents were plated on YPD agar plates and incubated overnight at 37°C. After incubation, *C. albicans* colony forming units (CFUs) were quantified using an automated colony counter (ProtoCOL 3; Synbiosis).

### Statistical analysis

Data are presented in bars as mean ± standard error of the mean (SEM) or scatterplots representing individual data points. Statistical tests used are detailed in the figure legends. *p* < 0.05 = *, *p* < 0.01 = **, and *p* < 0.001 = ***. Apart from gene expression and flowcytometry data, all data were analysed using Graphpad Prism v 9.

## Results

### AAT differentially impacts *C. albicans* clearance by mononuclear phagocytes

**Figure 1.**
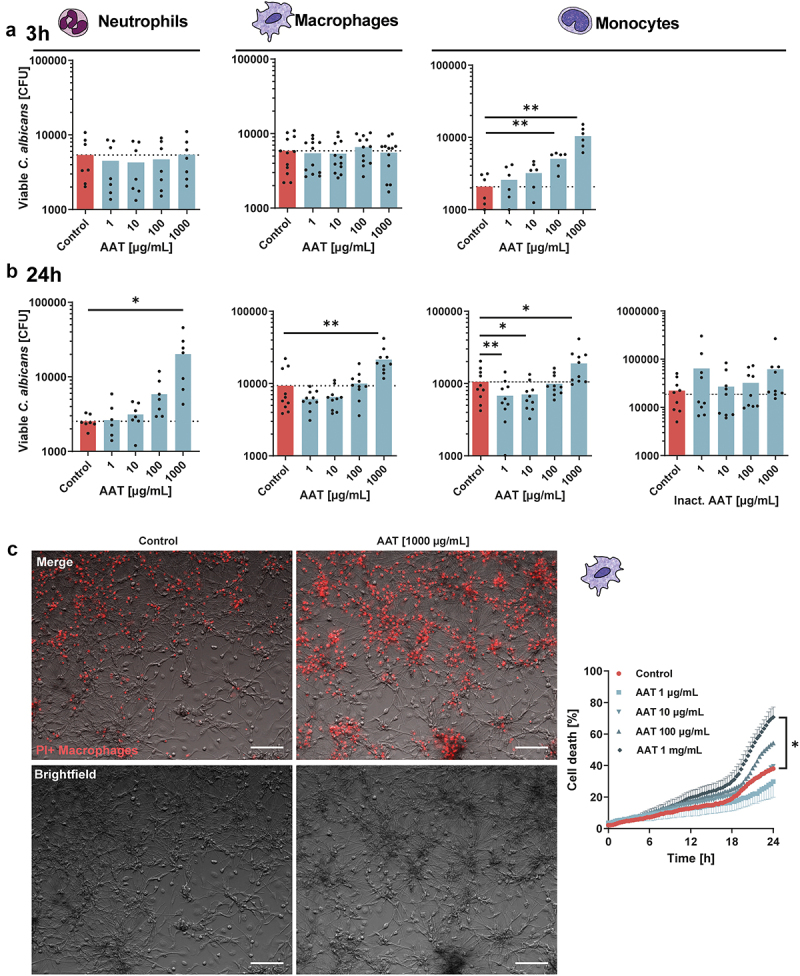
Serum protein AAT bidirectionally alters fungal killing efficiency in a cell type- and concentration-specific way. (a, b) CFU-based killing assays of *C. albicans* in presence of indicated concentrations of bioactive or heat-inactivated AAT. Live *C. albicans* SC5314 yeasts cells (MOI 1) were incubated with hMDMs (4 × 10^4^/well), neutrophils (1 × 10^5^/well) or monocytes (1 × 10^5^/well) and harvested for plating on YPD agar after (a) 3 h or (b) 24 h. Experiments were performed with neutrophils 3 h *n* = 7, 24 h *n* = 7; macrophages 3 h *n* = 12, macrophages 24 h *n* = 10; monocytes 3 h *n* = 6, 24 h *n* = 10, and 24 h inactivated AAT *n* = 9 donors. (c) Macrophage cell death quantification over time including representative merged and brightfield images after 24 h of human macrophages infected with *C. albicans* MOI 1 in presence or absence of AAT and PI to visualize cell death events (*n* = 6 donors). Scale bars equal 100 µm. Significant differences were determined by paired t-test as individual comparisons against the control group without AAT (a, b). Or two-way ANOVA (c). Data are displayed as the mean with individual biological replicates and significance levels *p < 0.05, **p < 0.01, ***p < 0.001.

Rapid fungal clearance is a prime role of the innate immune system during fungal infections [36]. Therefore, to characterize the role of AAT, the protein was supplemented to *in vitro* fungal killing assays. When adding AAT in a physiologically relevant range [37], no effects were observed on fungal survival during interactions with neutrophils or macrophages at early time points (3 h) (Figure 1(a)). However, the capacity of monocytes to control *C. albicans* at this early timepoint was significantly diminished with increasing AAT concentrations (Figure 1(a)). While investigating long-term (24 h) effects, AAT dose-dependently exerted a negative impact on fungal killing by neutrophils (Figure 1(b)). Similar to neutrophils, during the challenge with monocytes and macrophages, a significantly increased fungal survival was observed in the presence of high AAT concentrations after 24 h (Figure 1(b)). However, at low AAT concentrations fungal elimination by macrophages and particularly monocytes improved, a phenomenon that was not observed when heat-inactivated AAT was used (Figure 1(b)). At high concentrations, heat-inactivated AAT showed a trend towards compromising fungal clearance, yet not to the extent of bioactive AAT (Figure 1(b), Figure S1A). This could be attributable to the ability of particularly heat-inactivated AAT to serve as a nutrient source and promote fungal growth (Figure S1B) .

In macrophages, the immune escape of *C. albicans* is associated with the induction of cell lysis [38–40]. In parallel with the increased fungal survival in the presence of a high AAT concentration (1 mg/mL), an increased lysis of macrophages over time was observed (Figure 1(c), Figure S2). At the low concentrations of AAT (1 or 10 µg/mL), the improved fungal clearance in several donors was accompanied by a trend towards improved macrophage survival (Figure 1(c), Figures S2, S3).

Immune recognition and immune escape are tightly connected to changes in fungal cell biology and pathogenicity mechanisms [5,41]. Thus, we investigated specific effects of AAT on *C. albicans.*

### AAT binds *C. albicans* and triggers transcriptional responses

**Figure 2.**
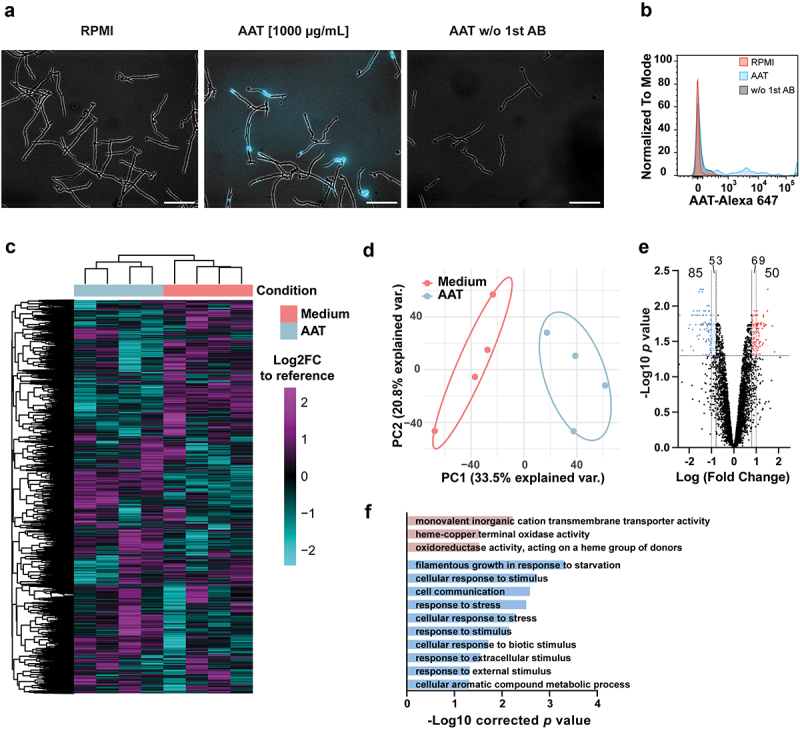
AAT binds to *C. albicans* and induces fungal transcriptional responses. (a) Representative immunofluorescence images of AAT on the surface of *C. albicans*. Data are representative for 3 biologically different fungal cultures. Scale bars equal 50 µm. (b) Overlaid flow cytometry histograms for AAT binding on fungal surface, *n* = 1. (C-F) Microarray data of *C. albicans* SC5314 treated with or without 100 µg/mL AAT at 3 h. (c) Heatmap of mRNA expression of *C. albicans* grown for 3 h in RPMI medium with or without 100 µg/mL AAT (*n* = 4) compared to a reference control of mid-log-phase YPD-grown *C. albicans*. Rows and columns are sorted by unsupervised hierarchal clustering using Euclidian distance. (d) Principal component analysis of global gene expression of *C. albicans* grown for 3 h in RPMI medium with or without 100 µg/mL AAT (*n* = 4) compared to the reference control (e) volcano plot of the Log2 fold change in gene expression between *C. albicans* grown for 3 h in RPMI medium with or without 100 µg/mL AAT. (f) GO-term enrichment analysis of differentially regulated genes (Log2 fold change > 0.8 or < -0.8 and *p* < 0.05) analyzed with the GO-term finder on the *Candida* Genome Database website.

A variety of human serum proteins, particularly complement proteins, have been shown to influence *C. albicans* pathogenicity mechanisms and immune escape [42–45]. In part, these host proteins interact with the fungus through surface binding. We observed that AAT also localizes to the surface of *C. albicans* (Figure 2(a,b)). Transcriptional profiling was performed to elucidate how the interaction with AAT changes *C. albicans* biology. *C. albicans* responded to the presence of AAT at the level of gene expression (Figure 2(c,d)), with a total of 135 genes differentially regulated (50 up-regulated and 85 down-regulated) compared to *C. albicans* grown in medium alone (Figure 2(e)). Gene ontology enrichment analysis did not reveal specific terms that could point towards increased virulence and thereby increased fungal escape from phagocytes (Figure 2(f)). However, the enrichment of the term “filamentous growth response in response to starvation” among the significantly downregulated genes caught our attention.

### AAT induces *C. albicans* filamentation

**Figure 3.**
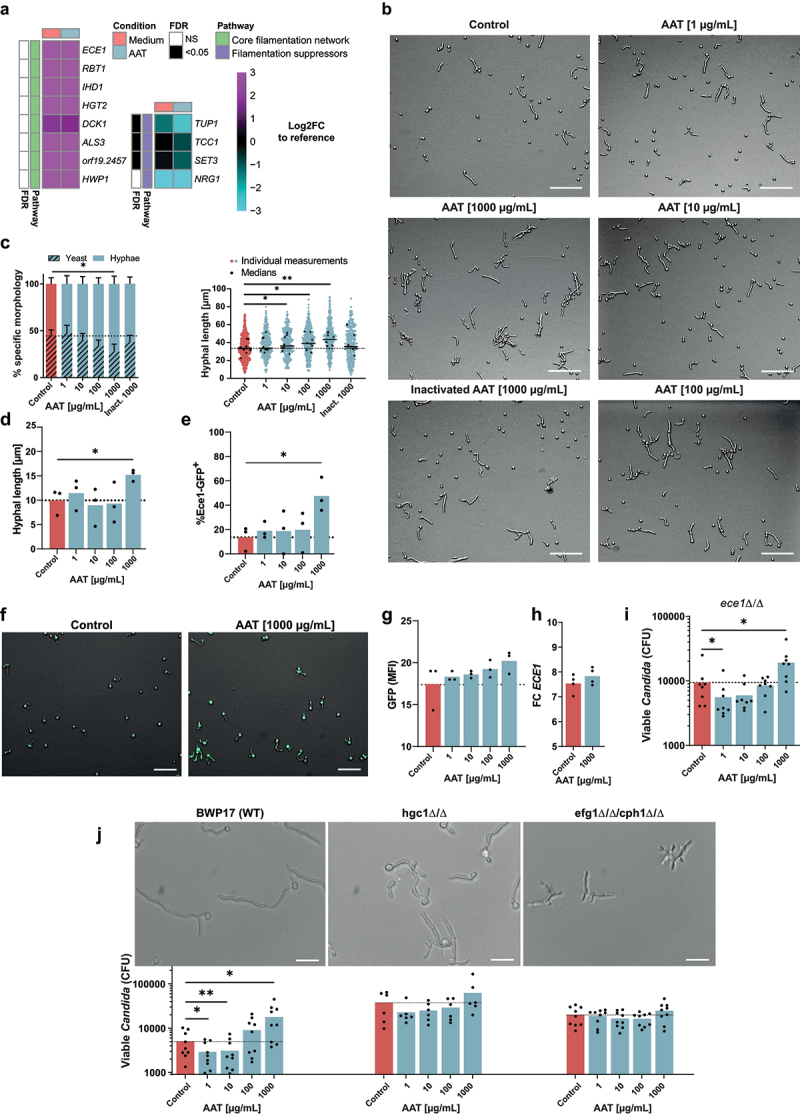
AAT increases *C. albicans* filamentation, which dictates the bidirectional impact of AAT on fungal killing. (a) Heatmap of the Log_2_FC in expression of core filamentation genes [46] and known filamentation repressors [47–49] compared to the reference control of mid-log-phase YPD-grown *C. albicans*. FDR calculated between the expression relative to the reference control of *C. albicans* grown for 3 h in RPMI medium with or without 100 µg/mL AAT. (b) Representative images of *C. albicans* grown in RPMI in the presence of increasing concentrations of AAT scale bars equal 50 µm. (c) Measurements of filamentation induction by increasing concentrations of AAT. Quantification of germination rate (number of hyphae/all fungal cells in view, displayed as mean + SEM; left) and hyphal length (right), displayed as mean (line) of medians of experiments (black dot) and single hyphae measurements (red/blue dots) after 3 h of culture at 37°C, 5% CO_2_. (d) Hyphal length of *C. albicans*, (e) quantification and (f) representative images of Ece1-GFP^+^ cells in PBS and the indicated AAT concentrations after 19 h at 37°C, 5% CO_2_. Scale bars equal 20 µm. (G) Mean fluorescence intensity of *p-Ece1*-GFP strain determined by microscopy after 3 h incubation in RPMI and indicated AAT concentrations at 37°C, 5% CO_2_. (h) *C. albicans ECE1* gene expression in the presence or absence of AAT (100 µg/mL; *n =* 4). (i) *C. albicans ece1Δ/Δ* killing by monocytes (*n =* 8 donors) in the presence or absence of AAT for 24 h quantified by remaining viable CFU. (j) Representative images WT (BWP17 Clp30), *hgc1Δ/Δ* and *efg1/cphΔ/Δ* morphology after 3 h growth in RPMI at 37°C, 5% CO_2._ scale bar equals 20 µm. *C. albicans* WT (*n =* 9 donors), *hgc1Δ/Δ* (*n =* 6 donors), or *efg1/cphΔ/Δ* (*n =* 9 donors) killing by monocytes in the presence or absence of AAT for 24 h quantified by remaining viable CFU. Significant differences were determined by paired t-test, as individual comparisons against the control group without AAT. If not mentioned otherwise, data are displayed as the mean with individual biological replicates and significance levels *p < 0.05, **p < 0.01, ***p < 0.001.

Initial assessment of the expression of the core-filamentation genes [46] did not reveal significant differences between the absence of AAT (Figure 3(a)). A closer look at the individual differentially regulated genes falling under the GO-term “filamentous growth response in response to starvation” revealed that this involved several known negative regulators of filamentation. *SET3* [47], as well as the filamentation repressor *TUP1* [48] and its co-repressor *TCC1* [49] were significantly downregulated 3 h after the fungus interacted with AAT (Figure 3(a)). These transcriptional changes were associated with a dose-dependent increase of *C. albicans* filamentation at 3 h in the presence of AAT in RPMI medium, yet heat-inactivated AAT did not induce filamentation (Figure 3(b,c)). Even under non-filamentation inducing conditions in PBS, a concentration of 1 mg/mL AAT was sufficient to induce filamentation (Figure 3(d,f)). This was associated with increased promotor activity of the hyphae-specific core filamentation gene *ECE1* [46] (Figure 3(e,f)). Under the hyphae-inducing condition of growth in RPMI, the AAT-mediated increase in filamentation was accompanied by a trend towards increased promotor activity of *ECE1* (Figure 3(g)), as well as a trend towards increased *ECE1* mRNA expression (Figure 3(h)). High *ECE1* mRNA expression generally correlates with a strong capacity to cause host-cell damage [50]. Nevertheless, it can be excluded that increased escape from phagocytes depends on the *ECE1-*encoded toxin candidalysin, as an *ece1Δ/Δ* mutant still showed an improved escape from monocytes at physiological (1 mg/mL) AAT concentrations (Figure 3(i)).

To assess whether the increased filamentation is directly linked to the improved *C. albicans* killing at low AAT concentrations and increased fungal escape at high AAT concentrations, two strains impaired in the yeast-to-hyphae transition were investigated. The *efg1Δ/Δ/cph1Δ/Δ* mutant is incapable of forming true hyphae under almost all conditions [21,51]. Not only was the *efg1Δ/Δ/cph1Δ/Δ* mutant unable to better escape monocyte killing at high AAT concentrations, the killing was also not improved at low AAT concentrations (Figure 3(j)). A *hgc1Δ/Δ* mutant cannot complete the hyphal program, yet expresses hyphae-associated genes [22]. Fitting to the phenotype of this mutant, a trend towards improved killing at low AAT concentrations and a trend towards more escape at high AAT concentrations was observed in some donors (Figure 3(j)).

We subsequently investigated how these transcriptional and morphological fungal adaptations in response to AAT could differentially influence fungal killing by mononuclear phagocytes.

### AAT-induced filamentation facilitates fungal immune escape

Filamentous growth is essential for the escape of *C. albicans* from mononuclear phagocytes [38,52–54]. This escape relies on several distinct mechanisms linked to *C. albicans* filamentation [6]: candidalysin-mediated host cell lysis [38], pyroptosis [25,39,40,55,56], hyphal extension [57], and mechanical forces [53,54].

**Figure 4.**
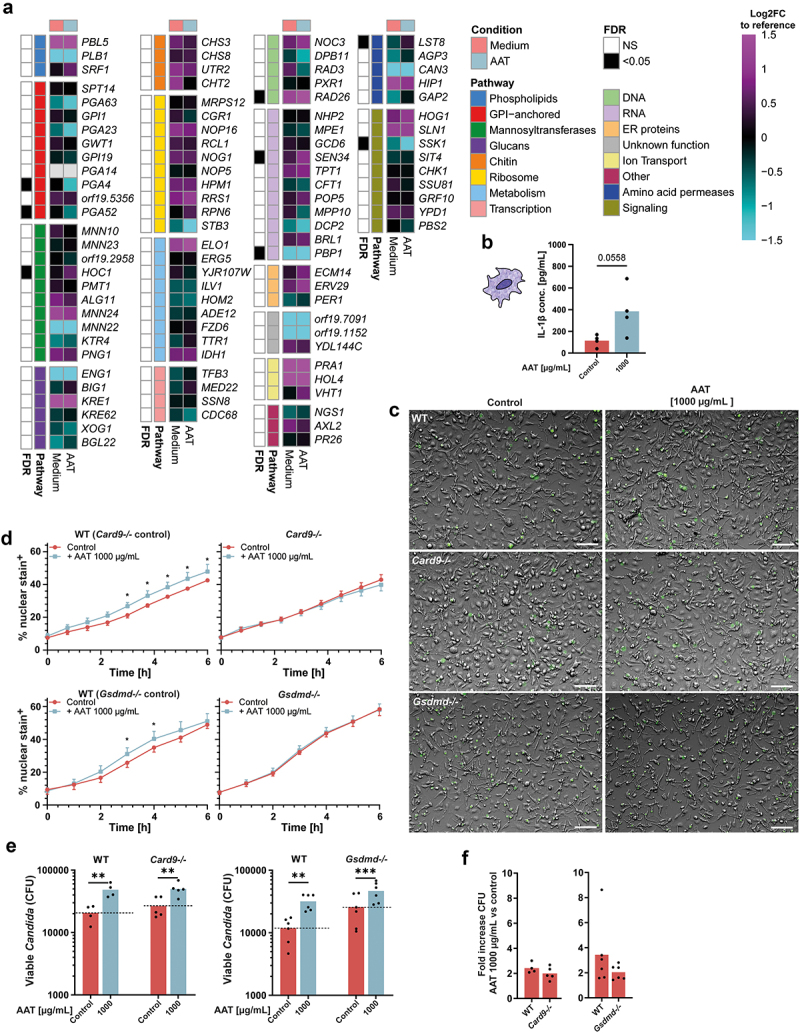
High AAT concentration facilitates *C. albicans* driven early induction of pyroptosis via CARD9 and GSDMD. (a) Heatmap of the Log_2_FC expression value of genes previously identified to be essential for pyroptosis [55] compared to the reference control of mid-log-phase YPD-grown *C. albicans*. FDR calculated between the expression relative to the reference control in *C. albicans* grown for 3 h in RPMI medium with or without 100 µg/mL AAT. (b) IL-1β release by MDMs (*n =* 4 donors) exposed to *C. albicans* for 24 h in the presence or absence of 1 mg/mL AAT. (c) Representative images and (d) quantification of live cell imaging of *C. albicans*-infected WT, *Card9-/-* and *Gsdmd-/-* BMDMs ±1 mg/mL AAT in the presence of Sytox Green to visualize cell death events, MOI 2.5. (e) Fungal survival after 24 h incubation with WT, *Card9-/-* and *Gsdmd-/-* BMDMs ±1 mg/mL AAT, determined by CFU plating, MOI 2.5. (f) Corresponding ratio of fungal survival between ±1 mg/mL AAT for the individual genotypes to visualize relative increase of fungal burden by AAT in those. Significant differences were determined by (b, e) paired t-test or (f) unpaired t-test, as individual comparisons against the control group without AAT. Data are displayed as the mean with individual biological replicates, except for (d), where RM two-way ANOVA was applied and the means + SEM of 4–6 biologically different cultures per group are displayed. Significance levels *p < 0.05, **p < 0.01, ***p < 0.001.

A variety of *C. albicans* genes have been associated with the induction of pyroptosis and escape from macrophages [55]. Interestingly, several genes (*HOC1, RAD26, SEN34*, and *LST8*) were up-regulated in response to AAT (Figure 4(a)). AAT is a potent inhibitor of the NLRP3 inflammasome [58]. Yet, 1 mg/mL AAT, enhanced the release of IL-1β, the hallmark cytokine for macrophage pyroptosis, by human macrophages after 24 h of exposure to live *C. albicans* (Figure 4(b)). To mechanistically unravel if pyroptosis is a contributing factor to AAT-driven fungal escape, we differentiated BMDMs from WT and *Gsdmd-/-* mice, which are deficient in undergoing classical pyroptosis [18,59]. We exposed those BMDMs to live *C. albicans* in the presence or absence of 1 mg/mL AAT and closely monitored their cell death kinetics by live cell imaging (Figure 4(c,d)). As pyroptosis can be a rather early event [25], we focused on the first 6 h of the interaction. Similar to human MDMs, the presence of 1 mg/mL AAT facilitated fungal escape by macrophage death in WT BMDMs, while in *Gsdmd-/-* BMDMs, addition of 1 mg/mL AAT did not change early death kinetics (Figure 4(c,d)). Similarly, the presence of 1 mg/mL AAT could not accelerate early macrophage death by *C. albicans* in *Card9-/-* BMDMs, substantiating active engagement of programmed cell death in BMDMs after AAT-boosted fungal recognition (Figure 4(c,d)). The high dose of AAT still significantly improved fungal survival in co-cultures with *Gsdmd-/-* and *Card9-/-* BMDMs, however with a, though not significantly different, lower efficacy compared to the WT (Figure 4(e,f)).

### AAT improves *C. albicans* recognition by inducing fungal cell wall remodeling

**Figure 5.**
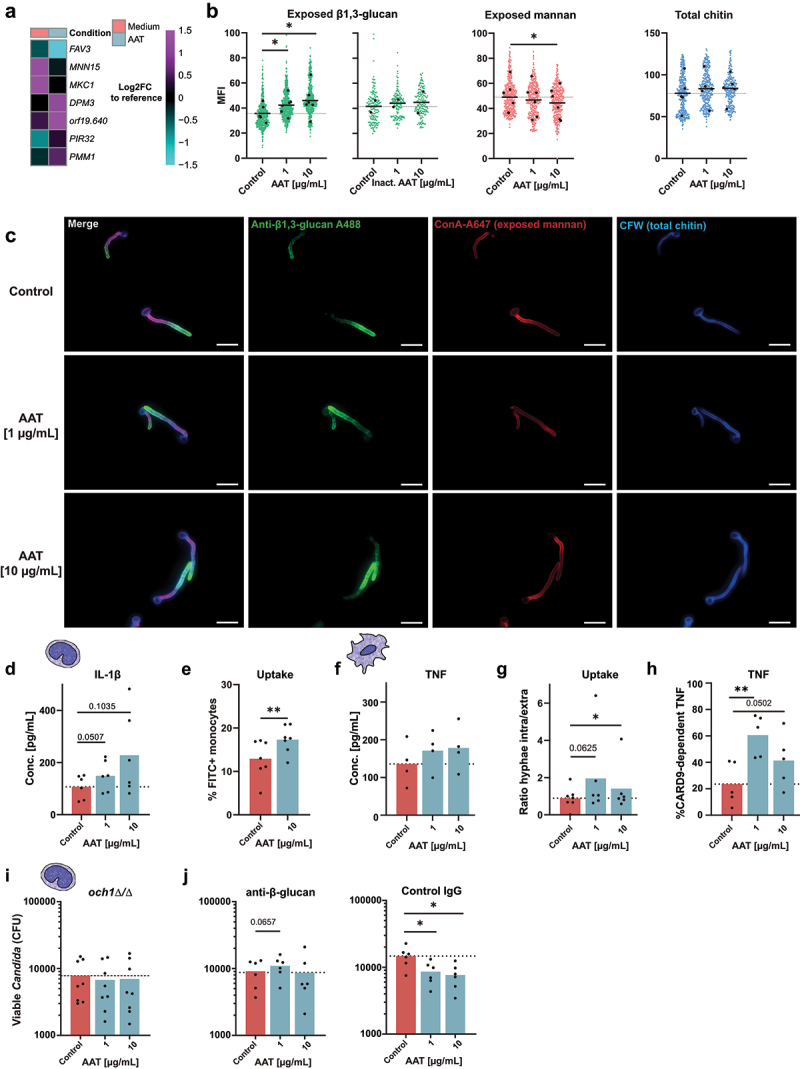
Low AAT concentrations induce sufficient hyphal formation to increase β-glucan exposure and promote fungal recognition and clearance. (a) Heatmap of genes involved in cell wall remodeling that were differentially expressed (Log2FC expression of <-0.8 or > 0.8 and an FDR < 0.05) between *C. albicans* grown for 3 h in RPMI medium with or without 100 µg/mL AAT relative to the reference control of mid-log-phase YPD-grown *C. albicans*. (b) Microscopy-based quantification of mean fluorescence intensity and (c) representative single channel and merge images at 40× magnification of cell wall stainings of AAT-treated *C. albicans* after 3 h incubation 37°C, 5% CO_2_ (green: β-glucan (*n* = 6), blue: chitin (*n* = 5), red: Mannan (*n* = 6)). (d-g) immunogenicity assessment of AAT-exposed *C. albicans*. (d) IL-1β release by human primary monocytes, 24 h stimulated with PFA-fixed *C. albicans*, which was grown ± AAT for 3 h before fixation. (e) Percentage of monocytes that engulfed FITC-stained *C. albicans* 2 hours after exposure in the presence or absence of 10 µg/mL AAT (f) TNF release from human MDMs, stimulated with viable *C. albicans* ± AAT for 3 h. (g) Microscopically determined ratio between intracellular and extracellular hyphae on human MDMs after 3 h co-culture ± AAT to quantify differences in fungal uptake by macrophages. (h) ELISA from supernatant of *Card9-/-* BMDMs co-cultured for 24 h with *C. albicans* (MOI 2.5) ± AAT. Concentrations of *Card9-/-* were normalized to the mean concentration of the corresponding WT group of the same AAT condition and the resulting values subtracted from 100% to provide the proportion of CARD9 dependent cytokine release at specific AAT conditions. (i) Killing of the *C. albicans och1Δ/Δ* deletion mutant (n *=* 8 donors), or (J) SC5314 in the presence of anti-β-glucan or control antibody (n *=* 6 donors) by monocytes in the presence or absence of AAT for 24 hours quantified by remaining viable CFUs. Significant differences were determined by paired t-test as individual comparisons against the control group without AAT. Data are displayed as the mean with individual biological replicates. Significance levels *p < 0.05, **p < 0.01, ***p < 0.001.

The fungal cell wall is the central immunogenic interface presented to innate immune cells and is undergoing drastic architectural changes during the *C. albicans* yeast-to-hyphae transition. This is accompanied by a recomposed exposure of pathogen-associated molecular patterns (PAMPs) [60], differentially affecting the recognition and killing of fungi by monocytes and macrophages [61]. Therefore, we examined the impact of AAT on PAMP exposure to understand, how low concentrations of AAT filamentation-dependently lead to improved *C. albicans* clearance. The transcriptional response of *C. albicans* to AAT indeed involved changes in genes that could suggest cell-wall remodelling (Figure 5(a)). The MAP kinase gene *MKC1* that regulates cell wall structure [62] was downregulated. Changes in the expression of the putative mannosyltransferase gene *MNN15* and the phosphomannomutase gene *PMM1* suggest changes in cell wall mannans. Further, increased expression of *orf19.640* encoding an integral membrane protein regulating β-glucan synthesis and a homolog to the *Saccharomyces cerevisiae* β-glucan synthesis gene *ScKEG1* [63] could suggest changes in β-glucan levels. To validate this, we assessed the exposure of cell wall mannan, β-1,3-glucan, as well as total chitin in response to the two AAT concentrations associated with improved fungal clearance (1 µg/mL and 10 µg/mL). While total chitin was not significantly altered by AAT, we observed a significant decrease of cell wall mannan at 10 µg/mL AAT. Mannan is a cell wall polysaccharide that can activate monocytes [61], but is also associated with structural masking of other immunogenic PAMPs such as the prototypic immune stimulatory fungal cell wall glycan β-1,3-glucan [61, 64] (Figure 5(b,c)). In line with the increased expression of *orf19.640* and decreased mannan levels, β-1,3-glucan exposure was significantly increased by both concentrations of AAT, though not by heat-inactivated AAT (Figure 5(b,c)). β-glucan is bound by several host receptors and is a key molecule to activate antifungal immune responses including cytokine release, phagocytosis and ROS production [65]. This led us to hypothesize that AAT alters fungal immunogenicity and increases recognition by driving β-glucan exposure. To verify this, we grew *C. albicans* in the presence or absence of AAT, fixed the fungus after removing AAT and exposed primary human monocytes to the inactivated pathogen. Interestingly, we observed an elevated IL-1β concentration in the supernatants of monocytes encountering the AAT-exposed fungus (Figure 5(d)), and monocytes showed an increased uptake of AAT-exposed *C. albicans* (Figure 5(e)). Similarly, human MDMs showed a trend to increased TNF release early after 3 h with live *C. albicans*, when low concentrations of AAT were present (Figure 5(f)), even though AAT is considered an inhibitor of the TNF releasing enzyme ADAM17 [8]. At the same time, MDMs displayed increased fungal uptake in the presence of low levels of AAT (Figure 5(g)). Connecting the recognition of β-glucan by its main receptor DECTIN-1 with the activation of NF-κB relies on the adaptor molecule CARD9 [66]. Adding AAT to WT and *Card9-/-* BMDM stimulated with *C. albicans* revealed that low AAT concentrations increased the dependency on CARD9 to release TNF (Figure 5(h)), supporting the idea of increased β-glucan-DECTIN-1 engagement in presence of AAT. To link the increased β-glucan exposure on the *C. albicans* surface with improved fungal clearance, the *och1Δ/Δ* mutant, which lacks an α-(1,6)-mannosyltransferase required to initiate the N-mannan structure that masks β-glucan [67] was used. In contrast to WT (Figure 1(b)), low AAT concentrations did not enhance the killing of *och1Δ/Δ* by monocytes (Figure 5(i)). Similarly, low AAT concentrations were unable to augment fungal killing by monocytes, when β-glucan was artificially masked by using an anti-β-glucan antibody (Figure 5(j)).

## Discussion

Here, we systematically investigated the impact of the pleiotropic immune modulator AAT in *C. albicans* host–pathogen interactions. We show that AAT binds to *C. albicans* and promotes its filamentation, thereby affecting the outcome of interactions with innate immune cells.

Upon exposure to AAT, *C. albicans* undergoes transcriptional reprogramming involving downregulation of the filamentous growth repressors *SET3, TUP1* [48] and *TCC1* [49]. This correlated with hyphae-driven escape from phagocytes, as fungal escape was not facilitated by a high dose of AAT in the yeast-locked mutant *efg1Δ/Δ/cph1Δ/Δ*. A *hgc1Δ/Δ* mutant, unable to progress filamentation [22], interestingly showed non-significant trends towards improved killing at low AAT concentrations and more escape at high AAT concentrations. Consequently, *C. albicans* filamentation seems key for the differential effects of AAT on fungal killing. The role of filamentation in escape from clearance by phagocytes has particularly been well characterized in macrophages [reviewed in 6]. Candidalysin is a major driver of fungal escape from macrophages [38–40]. Associated with the increased filamentation, we indeed observed a modest trend for increased expression of the candidalysin encoding gene *ECE1* in the presence of AAT. However, an *ece1Δ/Δ* strain still strongly benefitted from high AAT concentrations to escape monocytes, which disqualified candidalysin as the central factor for AAT-fuelled fungal escape.

When using heat-inactivated AAT it was evident that this could also foster fungal survival to some extent, but not to the magnitude of bioactive AAT. This may be attributed to the fact that high amounts of protein (either inactivated or bioactive) serve as a source of nutrients that enhances *C. albicans* growth. Nevertheless, the induction of filamentation was predominantly observed with bioactive AAT, while the growth benefit was higher with denatured protein. The result of the improved fungal survival may thus be the sum of increased filamentation, specific to bioactive AAT, and an increased fungal growth induced by the presence of either active or inactive protein.

Several *C. albicans* genes associated with pyroptosis and escape [55] were induced in response to AAT. Further, increased IL-1β release was observed with 1 mg/ml AAT, even though AAT is known to interfere with inflammasome activation [58]. The fungal cell wall remodelling in response to AAT also could have contributed to pyroptosis induction [68]. Deletion mutants of *PMM1*, one of the cell-wall remodelling genes up-regulated in response to AAT, are less capable of escaping macrophages [68]. The idea that active recognition of an AAT-remodelled cell wall promotes pyroptosis is supported by our observation that *Card9-/-* like *Gsdmd-/-* BMDMs are immune to early cell death enhancement in the presence of 1 mg/ml AAT. The signalling axis of β-glucan, DECTIN-1, SYK and CARD9 is a central pillar of inflammasome activation, as well as the release of inflammasome/pyroptosis-associated IL-1β and IL-18 in fungal infection and beyond [69–73]. In line with this, *C. albicans* mutants with defective β-glucan exposure cannot induce pyroptosis [25], although increased β-glucan exposure alone is insufficient to induce pyroptosis [56]. However, *Card9-/-* and *Gsdmd-/-* BMDMs displayed a partial rescue in the presence of 1 mg/ml AAT, especially when comparing fungal survival at later stages with WT BMDMs. Thus, the increased fungal escape at high concentrations of AAT can partially be explained early induction of pyroptosis in macrophages. However, the absence of a full recovery suggests additional fungal escape strategies beyond induction of pyroptosis that are supported by high doses of AAT. Particularly, longer growing hyphae pose direct problems for phagocytes. Hyphae are not efficiently engulfed [74], and upon engulfment, macrophages can form frustrated phagosomes with reduced microbicidal capacity [75]. Further, filamentation delays phagosome maturation [64], damages the phagosomal membrane [57], and facilitates the escape from phagocytes [25,38,52–55]. This underlines the importance of *C. albicans* filamentation for the effects observed in the presence of AAT. Yet, the redundancy of candidalysin and a moderate role for pyroptosis as escape strategy in the presence of AAT, rather propose a strong direct role for extending hyphae and the physical forces wielded by the process of filamentation.

A specific pattern of exposed fungal cell wall molecules may promote the engagement of programmed cell death in immune cells, yet the recognition of specific PAMPs is also vital to control *C. albicans* [5]. While β-glucan in the *C. albicans* cell wall is widely recognized as an important PAMP that drives fungal recognition mediated by DECTIN-1 and CARD9 [17,76–78] and complement receptor-3 [76,79], the cell wall mannans can interfere with the recognition of β-glucan [61]. Interestingly, mannan is important for recognition of *C. albicans* by monocytes and driving their TNF responses [61]. We observed that low dose exposure to AAT decreases mannan and increases β-glucan exposure on the *C. albicans* cell wall. These changes were paralleled by differential expression of genes involved in cell wall remodelling, such as suppression of the MAP kinase *MKC1*, the putative mannosyltransferase gene *MNN15*, and the increased expression of *orf19.640* involved in β-glucan synthesis. Increased β-glucan exposure was associated with an impaired early clearance by monocytes. However, at a later stage, both monocytes and macrophages seemed to benefit from increased β-glucan exposure, documented by improved fungal clearance at low AAT concentrations that were associated with accelerated fungal uptake and increased CARD9-dependent cytokine release. In line with this, antibody-mediated β-glucan masking neutralized the improved killing in the presence of AAT. Finally, AAT exhibited no beneficial effects on the killing of the *C. albicans och1Δ/Δ* mutant, which constitutively exposes β-glucan [50,67]. This underscores that the augmented fungal killing at low AAT concentrations is mediated through transcriptionally regulated fungal cell wall remodelling and thereby improved immune recognition.

It is intriguing how a serum protein can impact the outcome of *C. albicans*-phagocyte interactions by inducing fungal adaptations. Opportunistic pathogens such as *C. albicans* specialize to efficiently attune to their environment. These microbes not merely adapt to environmental stressors, but also sense environmental cues to prepare for upcoming threats, a phenomenon termed adaptive prediction [80,81]. Such responses can promote immune evasion and infection, but also commensalism [7]. Various host-relevant signals such as glucose concentrations [82], lactate, hypoxia, and iron limitation [78] have been associated with β-glucan masking and immune evasion. Besides, *C. albicans* was observed to alter filamentation in response to the immune mediators IFN, PGE2, and IL-17 [83,84], as well as the macrophage-derived protein PTMA [85]. Our data suggest that the plasma protein AAT is another host factor sensed by *C. albicans*, inducing adaptations that influence *C. albicans*-phagocyte interactions.

Collectively, we propose that at physiological serum concentrations, bioactive AAT increases both filamentation and growth to an extent that it exceeds the capacity of monocytes, macrophages, and neutrophils to control *C. albicans* over longer periods. In contrast, lower AAT concentrations, resembling tissue levels, enhanced monocyte and macrophage capacity to kill *C. albicans* as a result of transcriptionally regulated cell wall remodelling leading to more efficient immune recognition.

## Supplementary Material

Supplemental Material

## Data Availability

All metadata to replicate the findings of this study are provided in the methods, and data will be made available upon request unless this compromises blood donor anonymity. The microarray dataset generated in this study has been deposited in GEO Series accession number GSE165326 (http://www.ncbi.nlm.nih.gov/geo/query/acc.cgi?acc=GSE165326).

## References

[1] Wisplinghoff H, Ebbers J, Geurtz L, et al. Nosocomial bloodstream infections due to Candida spp. In the USA: species distribution, clinical features and antifungal susceptibilities. Int J Antimicrob Agents. 2014;43(1):78–19. doi: 10.1016/j.ijantimicag.2013.09.005. PubMed PMID: 24182454. 24182454

[2] d’Enfert C, Kaune AK, Alaban LR, et al. The impact of the fungus-host-microbiota interplay upon Candida albicans infections: current knowledge and new perspectives. FEMS Microbiol Rev. 2021;45(3). doi: 10.1093/femsre/fuaa060 PubMed PMID: 33232448; PubMed Central PMCID: PMC8100220. PMC8100220 33232448

[3] Brown GD, Denning DW, Gow NA, et al. Hidden killers: human fungal infections. Sci Transl Med. 2012;4(165):165rv13. doi: 10.1126/scitranslmed.3004404 PubMed PMID: 23253612. 23253612

[4] König A, Müller R, Mogavero S, et al. Fungal factors involved in host immune evasion, modulation and exploitation during infection. Cell Microbiol. 2021;23(1):e13272. doi: 10.1111/cmi.13272 32978997

[5] Erwig LP, Gow NA. Interactions of fungal pathogens with phagocytes. Nat Rev Microbiol. 2016;14(3):163–176. doi: 10.1038/nrmicro.2015.21 Epub 2016/02/09 PubMed PMID: 26853116. 26853116

[6] Austermeier S, Kasper L, Westman J, et al. I want to break free - macrophage strategies to recognize and kill Candida albicans, and fungal counter-strategies to escape. Curr Opin Microbiol. 2020;58:15–23. doi: 10.1016/j.mib.2020.05.007. Epub 20200627 PubMed PMID: 32599492. 32599492

[7] Brown AJP, Gow NAR, Warris A, et al. Memory in fungal pathogens promotes immune evasion, colonisation, and infection. Trends Microbiol. 2019;27(3):219–30. doi: 10.1016/j.tim.2018.11.001 Epub 2018/12/05 PubMed PMID: 30509563. 30509563

[8] Janciauskiene S, Welte T. Well-known and less well-known functions of alpha-1 antitrypsin. Its role in chronic obstructive pulmonary disease and other disease developments. Ann Am Thoracic Soc. 2016;13(Supplement_4):S280–S8. doi: 10.1513/AnnalsATS.201507-468KV PubMed PMID: 27564662. 27564662

[9] Miravitlles M. Alpha-1-antitrypsin and other proteinase inhibitors. Curr Opin Pharmacol. 2012;12(3):309–14. doi: 10.1016/j.coph.2012.02.004 PubMed PMID: 22365503. 22365503

[10] Lewis EC. Expanding the clinical indications for α1-antitrypsin therapy. Mol Med. 2012;18(6):957–970. doi: 10.2119/molmed.2011.00196 Epub 2012/05/29 PubMed PMID: 22634722; PubMed Central PMCID: PMC3459478. 22634722 PMC3459478

[11] Jonigk D, Al-Omari M, Maegel L, et al. Anti-inflammatory and immunomodulatory properties of alpha1-antitrypsin without inhibition of elastase. Proc Natl Acad Sci U S A. 2013;110(37):15007–15012. doi: 10.1073/pnas.1309648110 PubMed PMID: 23975926; PubMed Central PMCID: PMC3773761. 23975926 PMC3773761

[12] Wewers MD, Crystal RG. Alpha-1 antitrypsin augmentation therapy. COPD: J Chronic Obstructive Pulmonary Dis. 2013;10 Suppl 1(sup1):64–67. doi: 10.3109/15412555.2013.764402 PubMed PMID: 23527997. 23527997

[13] Magenau JM, Goldstein SC, Peltier D, et al. alpha1-antitrypsin infusion for treatment of steroid-resistant acute graft-versus-host disease. Blood. 2018;131(12):1372–1379. doi: 10.1182/blood-2017-11-815746 Epub 2018/02/14 PubMed PMID: 29437593; PubMed Central PMCID: PMC5865235. 29437593 PMC5865235

[14] Marcondes AM, Hockenbery D, Lesnikova M, et al. Response of steroid-refractory acute GVHD to alpha1-antitrypsin. Biol Blood Marrow Transplant. 2016;22(9):1596–1601. doi: 10.1016/j.bbmt.2016.05.011 Epub 2016/05/26 PubMed PMID: 27223109. 27223109

[15] Matzaraki V, Gresnigt MS, Jaeger M, et al. An integrative genomics approach identifies novel pathways that influence candidaemia susceptibility. PloS One. 2017;12(7):e0180824. doi: 10.1371/journal.pone.0180824 Epub 2017/07/21 PubMed PMID: 28727728; PubMed Central PMCID: PMC5519064. 28727728 PMC5519064

[16] Bruno M, Dewi IMW, Matzaraki V, et al. Comparative host transcriptome in response to pathogenic fungi identifies common and species-specific transcriptional antifungal host response pathways. Comput Struct Biotechnol J. 2021;19:647–663. doi: 10.1016/j.csbj.2020.12.036 Epub 2021/01/30 PubMed PMID: 33510868; PubMed Central PMCID: PMC7817431. 33510868 PMC7817431

[17] Gross O, Gewies A, Finger K, et al. Card9 controls a non-TLR signalling pathway for innate anti-fungal immunity. Nature. 2006;442(7103):651–6. doi: 10.1038/nature04926. Epub 2006/07/25 PubMed PMID: 16862125. 16862125

[18] Kayagaki N, Stowe IB, Lee BL, et al. Caspase-11 cleaves gasdermin D for non-canonical inflammasome signalling. Nature. 2015;526(7575):666–71. doi: 10.1038/nature15541 26375259

[19] Gillum AM, Tsay EY, Kirsch DR. Isolation of the Candida albicans gene for orotidine-5’-phosphate decarboxylase by complementation of S. cerevisiae ura3 and E. coli pyrF mutations. Mol Gen Genet. 1984;198(2):179–182. doi: 10.1007/BF00328721 Epub 1984/01/01 PubMed PMID: 6394964. 6394964

[20] Zakikhany K, Naglik JR, Schmidt-Westhausen A, et al. In vivo transcript profiling of Candida albicans identifies a gene essential for interepithelial dissemination. Cell Microbiol. 2007;9(12):2938–54. doi: 10.1111/j.1462-5822.2007.01009.x PubMed PMID: 17645752. 17645752

[21] Wartenberg A, Linde J, Martin R, et al. Microevolution of Candida albicans in macrophages restores filamentation in a nonfilamentous mutant. PLoS Genet. 2014;10(12):e1004824. doi: 10.1371/journal.pgen.1004824 25474009 PMC4256171

[22] Zheng X, Wang Y, Wang Y. Hgc1, a novel hypha-specific G1 cyclin-related protein regulates Candida albicans hyphal morphogenesis. EMBO J. 2004;23(8):1845–56. doi: 10.1038/sj.emboj.7600195 Epub 2004/04/09 PubMed PMID: 15071502; PubMed Central PMCID: PMC394249. 15071502 PMC394249

[23] Moyes DL, Wilson D, Richardson JP, et al. Candidalysin is a fungal peptide toxin critical for mucosal infection. Nature. 2016;532(7597):64–8. doi: 10.1038/nature17625. PubMed PMID: 27027296; PubMed Central PMCID: PMC4851236. 27027296 PMC4851236

[24] Bates S, Bleddyn Hughes H, Munro CA, et al. Outer chain N-glycans are required for cell wall integrity and virulence of Candida albicans. J Biol Chem. 2006;281(1):90–8. doi: 10.1074/jbc.M510360200 16263704

[25] Uwamahoro N, Verma-Gaur J, Shen HH, et al. The pathogen Candida albicans hijacks pyroptosis for escape from macrophages. MBio. 2014;5(2):e00003–14. doi: 10.1128/mBio.00003-14. Epub 2014/03/29 PubMed PMID: 24667705; PubMed Central PMCID: PMC3977349. 24667705 PMC3977349

[26] Schindelin J, Arganda-Carreras I, Frise E, et al. Fiji: an open-source platform for biological-image analysis. Nat Methods. 2012;9(7):676–82. doi: 10.1038/nmeth.2019. Epub 2012/06/30 PubMed PMID: 22743772; PubMed Central PMCID: PMC3855844. 22743772 PMC3855844

[27] Wachtler B, Wilson D, Haedicke K, et al. From attachment to damage: defined genes of Candida albicans mediate adhesion, invasion and damage during interaction with oral epithelial cells. PloS One. 2011;6(2):e17046. doi: 10.1371/journal.pone.0017046 Epub 2011/03/17 PubMed PMID: 21407800; PubMed Central PMCID: PMC3044159. 21407800 PMC3044159

[28] Luttich A, Brunke S, Hube B. Isolation and amplification of fungal RNA for microarray analysis from host samples. Methods Mol Biol. 2012;845:411–421. doi: 10.1007/978-1-61779-539-8_28. PubMed PMID: 22328391. 22328391

[29] Hebecker B, Vlaic S, Conrad T, et al. Dual-species transcriptional profiling during systemic candidiasis reveals organ-specific host-pathogen interactions. Sci Rep. 2016;6:36055. doi: 10.1038/srep36055 PubMed PMID: 27808111; PubMed Central PMCID: PMC5093689. 27808111 PMC5093689

[30] Huber W, Carey VJ, Gentleman R, et al. Orchestrating high-throughput genomic analysis with Bioconductor. Nat Methods. 2015;12(2):115–21. doi: 10.1038/nmeth.3252. Epub 2015/01/31 PubMed PMID: 25633503; PubMed Central PMCID: PMC4509590. 25633503 PMC4509590

[31] Ritchie ME, Phipson B, Wu D, et al. Limma powers differential expression analyses for RNA-sequencing and microarray studies. Nucleic Acids Res. 2015;43(7):e47. doi: 10.1093/nar/gkv007 Epub 2015/01/22 PubMed PMID: 25605792; PubMed Central PMCID: PMC4402510. 25605792 PMC4402510

[32] Kolde R. Pheatmap: pretty heatmaps. R package. 2015;1(7):790

[33] Skrzypek MS, Binkley J, Binkley G, et al. The Candida Genome Database (CGD): incorporation of assembly 22, systematic identifiers and visualization of high throughput sequencing data. Nucleic Acids Res. 2017;45(D1):D592–D6. doi: 10.1093/nar/gkw924 Epub 20161013 PubMed PMID: 27738138; PubMed Central PMCID: PMC5210628. 27738138 PMC5210628

[34] Nogueira MF, Istel F, Jenull S, et al. Quantitative analysis of candida cell wall components by flow cytometry with triple-fluorescence staining. J Microbiol Modern Tech. 2017;2(1). doi: 10.15744/2575-5498.2.101

[35] Santos EO, Azzolini AE, Lucisano-Valim YM. Optimization of a flow cytometric assay to evaluate the human neutrophil ability to phagocytose immune complexes via fcgamma and complement receptors. J Pharmacol Toxicol Methods. 2015;72:67–71. doi: 10.1016/j.vascn.2014.10.005. PubMed PMID: 25450839. 25450839

[36] Netea MG, Joosten LA, van der Meer JW, et al. Immune defence against Candida fungal infections. Nat Rev Immunol. 2015;15(10):630–642. doi: 10.1038/nri3897 PubMed PMID: 26388329. 26388329

[37] Ter Horst R, Jaeger M, Smeekens SP, et al. Host and environmental factors influencing individual human cytokine responses. Cell. 2016;167(4):1111–24 e13. doi: 10.1016/j.cell.2016.10.018 Epub 2016/11/05 PubMed PMID: 27814508; PubMed Central PMCID: PMC5787854. 27814508 PMC5787854

[38] Kasper L, Konig A, Koenig PA, et al. The fungal peptide toxin Candidalysin activates the NLRP3 inflammasome and causes cytolysis in mononuclear phagocytes. Nat Commun. 2018;9(1):4260. doi: 10.1038/s41467-018-06607-1. Epub 2018/10/17 PubMed PMID: 30323213; PubMed Central PMCID: PMC6189146. 30323213 PMC6189146

[39] Ding X, Kambara H, Guo R, et al. Inflammasome-mediated GSDMD activation facilitates escape of Candida albicans from macrophages. Nat Commun. 2021;12(1):6699. doi: 10.1038/s41467-021-27034-9. Epub 20211118 PubMed PMID: 34795266. 34795266 PMC8602704

[40] Olivier FAB, Hilsenstein V, Weerasinghe H, et al. The escape of Candida albicans from macrophages is enabled by the fungal toxin candidalysin and two host cell death pathways. Cell Rep. 2022;40(12):111374. doi: 10.1016/j.celrep.2022.111374 PubMed PMID: 36130496. 36130496

[41] Hopke A, Brown AJP, Hall RA, et al. Dynamic fungal cell wall architecture in stress adaptation and immune evasion. Trends Microbiol. 2018;26(4):284–95. doi: 10.1016/j.tim.2018.01.007 29452950 PMC5869159

[42] Behrens NE, Lipke PN, Pilling D, et al. Serum amyloid P component binds fungal surface amyloid and decreases human macrophage phagocytosis and secretion of inflammatory cytokines. MBio. 2019;10(2). doi: 10.1128/mBio.00218-19 Epub 2019/03/14 PubMed PMID: 30862745; PubMed Central PMCID: PMC6414697. PMC6414697 30862745

[43] Pinsky M, Roy U, Moshe S, et al. Human serum albumin facilitates heme-iron utilization by Fungi. MBio. 2020;11(2). doi: 10.1128/mBio.00607-20 Epub 2020/04/23 PubMed PMID: 32317324; PubMed Central PMCID: PMC7175094. PMC7175094 32317324

[44] Luo S, Poltermann S, Kunert A, et al. Immune evasion of the human pathogenic yeast Candida albicans: Pra1 is a factor H, FHL-1 and plasminogen binding surface protein. Mol Immunol. 2009;47(2–3):541–50. doi: 10.1016/j.molimm.2009.07.017 Epub 2009/10/24 PubMed PMID: 19850343. 19850343

[45] Lopez CM, Wallich R, Riesbeck K, et al. Candida albicans uses the surface protein Gpm1 to attach to human endothelial cells and to keratinocytes via the adhesive protein vitronectin. PloS One. 2014;9(3):e90796. doi: 10.1371/journal.pone.0090796 Epub 20140313 PubMed PMID: 24625558; PubMed Central PMCID: PMC3953207. 24625558 PMC3953207

[46] Martin R, Albrecht-Eckardt D, Brunke S, et al. A core filamentation response network in Candida albicans is restricted to eight genes. PloS One. 2013;8(3):e58613. doi: 10.1371/journal.pone.0058613 Epub 2013/03/22 PubMed PMID: 23516516; PubMed Central PMCID: PMC3597736. 23516516 PMC3597736

[47] Hnisz D, Majer O, Frohner IE, et al. The Set3/Hos2 histone deacetylase complex attenuates cAMP/PKA signaling to regulate morphogenesis and virulence of Candida albicans. PLOS Pathog. 2010;6(5):e1000889. doi: 10.1371/journal.ppat.1000889 Epub 20100513 PubMed PMID: 20485517; PubMed Central PMCID: PMC2869326. 20485517 PMC2869326

[48] Braun BR, Johnson AD. Control of filament formation in Candida albicans by the transcriptional repressor TUP1. Science. 1997;277(5322):105–9. doi: 10.1126/science.277.5322.105 Epub 1997/07/04 PubMed PMID: 9204892. 9204892

[49] Kaneko A, Umeyama T, Utena-Abe Y, et al. Tcc1p, a novel protein containing the tetratricopeptide repeat motif, interacts with Tup1p to regulate morphological transition and virulence in Candida albicans. Eukaryot Cell. 2006;5(11):1894–905. doi: 10.1128/EC.00151-06. Epub 2006/09/26 PubMed PMID: 16998076; PubMed Central PMCID: PMC1694794. 16998076 PMC1694794

[50] Mogavero S, Sauer FM, Brunke S, et al. Candidalysin delivery to the invasion pocket is critical for host epithelial damage induced by Candida albicans. Cell Microbiol. 2021;23(10):e13378. doi: 10.1111/cmi.13378 Epub 20210720 PubMed PMID: 34245079; PubMed Central PMCID: PMC8460606. 34245079 PMC8460606

[51] Lo HJ, Kohler JR, DiDomenico B, et al. Nonfilamentous C. albicans mutants are avirulent. Cell. 1997;90(5):939–949. doi: 10.1016/S0092-8674(00)80358-X PubMed PMID: 9298905. 9298905

[52] Tucey TM, Verma J, Harrison PF, et al. Glucose homeostasis is important for immune cell viability during Candida Challenge and Host survival of systemic fungal infection. Cell Metab. 2018;27(5):988–1006 e7. doi: 10.1016/j.cmet.2018.03.019 PubMed PMID: 29719235. 29719235 PMC6709535

[53] McKenzie CG, Koser U, Lewis LE, et al. Contribution of Candida albicans cell wall components to recognition by and escape from murine macrophages. Infect Immun. 2010;78(4):1650–8. doi: 10.1128/IAI.00001-10. Epub 2010/02/04 PubMed PMID: 20123707; PubMed Central PMCID: PMC2849426. 20123707 PMC2849426

[54] Ghosh S, Navarathna DH, Roberts DD, et al. Arginine-induced germ tube formation in Candida albicans is essential for escape from murine macrophage line RAW 264.7. Infect Immun. 2009;77(4):1596–1605. doi: 10.1128/IAI.01452-08 Epub 2009/02/04 PubMed PMID: 19188358; PubMed Central PMCID: PMC2663133. 19188358 PMC2663133

[55] O’Meara TR, Duah K, Guo CX, et al. High-throughput screening identifies genes required for Candida albicans induction of macrophage pyroptosis. MBio. 2018;9(4). doi: 10.1128/mBio.01581-18 Epub 20180821 PubMed PMID: 30131363; PubMed Central PMCID: PMC6106084. PMC6106084 30131363

[56] Wellington M, Koselny K, Sutterwala FS, et al. Candida albicans triggers NLRP3-mediated pyroptosis in macrophages. Eukaryot Cell. 2014;13(2):329–40. doi: 10.1128/EC.00336-13. Epub 2014/01/01 PubMed PMID: 24376002; PubMed Central PMCID: PMC3910967. 24376002 PMC3910967

[57] Westman J, Moran G, Mogavero S, et al. Candida albicans hyphal expansion causes phagosomal membrane damage and luminal alkalinization. MBio. 2018;9(5). doi: 10.1128/mBio.01226-18 Epub 2018/09/13 PubMed PMID: 30206168; PubMed Central PMCID: PMC6134096. PMC6134096 30206168

[58] Ebrahimi T, Rust M, Kaiser SN, et al. alpha1-antitrypsin mitigates NLRP3-inflammasome activation in amyloid beta1-42-stimulated murine astrocytes. J Neuroinflammation. 2018;15(1):282. doi: 10.1186/s12974-018-1319-x Epub 20180927 PubMed PMID: 30261895; PubMed Central PMCID: PMC6158809. 30261895 PMC6158809

[59] Shi J, Zhao Y, Wang K, et al. Cleavage of GSDMD by inflammatory caspases determines pyroptotic cell death. Nature. 2015;526(7575):660–5. doi: 10.1038/nature15514. Epub 2015/09/17 PubMed PMID: 26375003. 26375003

[60] Lowman DW, Greene RR, Bearden DW, et al. Novel structural features in Candida albicans hyphal glucan provide a basis for differential innate immune recognition of hyphae versus yeast. J Biol Chem. 2014;289(6):3432–43. doi: 10.1074/jbc.M113.529131 Epub 2013/12/18 PubMed PMID: 24344127; PubMed Central PMCID: PMC3916545. 24344127 PMC3916545

[61] Yadav B, Mora-Montes HM, Wagener J, et al. Differences in fungal immune recognition by monocytes and macrophages: N-mannan can be a shield or activator of immune recognition. Cell Surf. 2020;6:100042. doi: 10.1016/j.tcsw.2020.100042 Epub 20200721 PubMed PMID: 33364531; PubMed Central PMCID: PMC7750734. 33364531 PMC7750734

[62] Navarro-Garcia F, Alonso-Monge R, Rico H, et al. A role for the MAP kinase gene MKC1 in cell wall construction and morphological transitions in Candida albicans. Microbiology. 1998;144(Pt 2):411–424. doi: 10.1099/00221287-144-2-411 Epub 1998/03/11 PubMed PMID: 9493378. 9493378

[63] Nakamata K, Kurita T, Bhuiyan MS, et al. KEG1/YFR042w encodes a novel Kre6-binding endoplasmic reticulum membrane protein responsible for beta-1,6-glucan synthesis in Saccharomyces cerevisiae. J Biol Chem. 2007;282(47):34315–34324. doi: 10.1074/jbc.M706486200 Epub 2007/09/26 PubMed PMID: 17893149. 17893149

[64] Bain JM, Louw J, Lewis LE, et al. Candida albicans hypha formation and Mannan Masking of β-glucan inhibit macrophage phagosome maturation. MBio. 2014;5(6):e01874. doi: 10.1128/mBio.01874-14 Epub 2014/12/04 PubMed PMID: 25467440; PubMed Central PMCID: PMC4324242. 25467440 PMC4324242

[65] Goodridge HS, Wolf AJ, Underhill DM. Beta-glucan recognition by the innate immune system. Immunol Rev. 2009;230(1):38–50. doi: 10.1111/j.1600-065X.2009.00793.x PubMed PMID: 19594628; PubMed Central PMCID: PMC6618291. 19594628 PMC6618291

[66] Reid DM, Gow NA, Brown GD. Pattern recognition: recent insights from dectin-1. Curr Opin Immunol. 2009;21(1):30–7. doi: 10.1016/j.coi.2009.01.003 Epub 20090214 PubMed PMID: 19223162; PubMed Central PMCID: PMC2684021. 19223162 PMC2684021

[67] Wheeler RT, Fink GR. A drug-sensitive genetic network masks fungi from the immune system. PLOS Pathog. 2006;2(4):e35. doi: 10.1371/journal.ppat.0020035 Epub 2006/05/03. 16652171 PMC1447670

[68] O’Meara TR, Veri AO, Ketela T, et al. Global analysis of fungal morphology exposes mechanisms of host cell escape. Nat Commun. 2015;6:6741. doi: 10.1038/ncomms7741. Epub 20150331 PubMed PMID: 25824284; PubMed Central PMCID: PMC4382923. 25824284 PMC4382923

[69] Drummond RA, Swamydas M, Oikonomou V, et al. CARD9(+) microglia promote antifungal immunity via IL-1β- and CXCL1-mediated neutrophil recruitment. Nat Immunol. 2019;20(5):559–570. doi: 10.1038/s41590-019-0377-2 Epub 2019/04/19 PubMed PMID: 30996332; PubMed Central PMCID: PMC6494474. 30996332 PMC6494474

[70] Ganesan S, Rathinam VAK, Bossaller L, et al. Caspase-8 modulates dectin-1 and complement receptor 3-driven IL-1β production in response to β-glucans and the fungal pathogen, Candida albicans. J Immunol. 2014;193(5):2519–2530. doi: 10.4049/jimmunol.1400276 Epub 2014/07/27 PubMed PMID: 25063877; PubMed Central PMCID: PMC4134963. 25063877 PMC4134963

[71] Gringhuis SI, Kaptein TM, Wevers BA, et al. Dectin-1 is an extracellular pathogen sensor for the induction and processing of IL-1β via a noncanonical caspase-8 inflammasome. Nat Immunol. 2012;13(3):246–54. doi: 10.1038/ni.2222. Epub 2012/01/24 PubMed PMID: 22267217. 22267217

[72] Malik A, Sharma D, Malireddi RKS, et al. SYK-CARD9 signaling axis promotes gut fungi-mediated inflammasome activation to restrict colitis and colon cancer. Immunity. 2018;49(3):515–30.e5. doi: 10.1016/j.immuni.2018.08.024 Epub 2018/09/21 PubMed PMID: 30231985; PubMed Central PMCID: PMC6541497. 30231985 PMC6541497

[73] Rhoads JP, Lukens JR, Wilhelm AJ, et al. Oxidized low-density lipoprotein immune complex priming of the Nlrp3 inflammasome involves TLR and FcγR cooperation and is dependent on CARD9. J Immunol. 2017;198(5):2105–14. doi: 10.4049/jimmunol.1601563 Epub 2017/01/29 PubMed PMID: 28130494; PubMed Central PMCID: PMC5318843. 28130494 PMC5318843

[74] Lewis LE, Bain JM, Lowes C, et al. Stage specific assessment of Candida albicans phagocytosis by macrophages identifies cell wall composition and morphogenesis as key determinants. PLOS Pathog. 2012;8(3):e1002578. doi: 10.1371/journal.ppat.1002578 Epub 2012/03/23 PubMed PMID: 22438806; PubMed Central PMCID: PMC3305454. 22438806 PMC3305454

[75] Maxson ME, Naj X, O’Meara TR, et al. Integrin-based diffusion barrier separates membrane domains enabling the formation of microbiostatic frustrated phagosomes. Elife. 2018;7:e34798. doi: 10.7554/eLife.34798 Epub 2018/03/20. 29553370 PMC5897098

[76] Gow NAR, van de Veerdonk FL, Brown AJP, et al. Candida albicans morphogenesis and host defence: discriminating invasion from colonization. Nature Rev Microbiol. 2012;10(2):112–22. doi: 10.1038/nrmicro2711 PMC3624162 22158429

[77] Brown GD, Taylor PR, Reid DM, et al. Dectin-1 is a Major β-glucan receptor on macrophages. J Exp Med. 2002;196(3):407–412. doi: 10.1084/jem.20020470 12163569 PMC2193936

[78] Pradhan A, Avelar GM, Bain JM, et al. Non-canonical signalling mediates changes in fungal cell wall PAMPs that drive immune evasion. Nat Commun. 2019;10(1):5315. doi: 10.1038/s41467-019-13298-9. Epub 2019/11/24 PubMed PMID: 31757950; PubMed Central PMCID: PMC6876565. 31757950 PMC6876565

[79] Thornton BP, Vetvicka V, Pitman M, et al. Analysis of the sugar specificity and molecular location of the beta-glucan-binding lectin site of complement receptor type 3 (CD11b/CD18). J Immunol. 1996;156(3):1235–46. doi: 10.4049/jimmunol.156.3.1235 8558003

[80] Brunke S, Hube B. Adaptive prediction as a strategy in microbial infections. PLOS Pathog. 2014;10(10):e1004356. doi: 10.1371/journal.ppat.1004356 Epub 2014/10/03. 25275642 PMC4183746

[81] Mitchell A, Romano GH, Groisman B, et al. Adaptive prediction of environmental changes by microorganisms. Nature. 2009;460(7252):220–4. doi: 10.1038/nature08112. Epub 2009/06/19 PubMed PMID: 19536156. 19536156

[82] Rodaki A, Bohovych IM, Enjalbert B, et al. Glucose promotes stress resistance in the fungal pathogen Candida albicans. Mol Biol Cell. 2009;20(22):4845–4855. doi: 10.1091/mbc.E09-01-0002 Epub 2009/09/18 PubMed PMID: 19759180; PubMed Central PMCID: PMC2777113. 19759180 PMC2777113

[83] Kalo-Klein A, Witkin SS. Prostaglandin E2 enhances and Gamma Interferon inhibits germ tube formation in Candida albicans infection and immunity. Infect Immun. 1989;58(1):260–262. doi: 10.1128/iai.58.1.260-262.1990 PMC258440 2152888

[84] Zelante T, Iannitti RG, De Luca A, et al. Sensing of mammalian IL-17A regulates fungal adaptation and virulence. Nat Commun. 2012;3:683. doi: 10.1038/ncomms1685 PubMed PMID: 22353714. 22353714

[85] Case NT, Duah K, Larsen B, et al. The macrophage-derived protein PTMA induces filamentation of the human fungal pathogen Candida albicans. Cell Rep. 2021;36(8):109584. doi: 10.1016/j.celrep.2021.109584 Epub 2021/08/26 PubMed PMID: 34433036; PubMed Central PMCID: PMC8454912. 34433036 PMC8454912

