# Supplementary material for: Alpha1-antitrypsin impacts innate host–pathogen interactions with Candida albicans by stimulating fungal filamentation
Source: Virulence. 2024 Mar 22;15(1):2333367. doi: 10.1080/21505594.2024.2333367 (PMC11008552; doi:10.1080/21505594.2024.2333367)
Supplement: Supplemental Material [file KVIR_A_2333367_SM4061.zip › Supplemental_data.docx]

**Supplemental data:**

**Alpha1-antitrypsin impacts innate host-pathogen interactions with *Candida albicans* by stimulating fungal filamentation**

Martin Jaeger^a,b,c*^, Axel Dietschmann^d*^ Sophie Austermeier^e^, Sude Dinçer^d^, Pauline Porschitz^d^, Larsen Vornholz^h^, Ralph J.A. Maas^a,f^, Evelien G.G. Sprenkeler^b^, Jürgen Ruland^h^, Stefan Wirtz^i^, Tania Azam^a^, Leo A.B. Joosten^b^, Bernhard Hube^e,g^, Mihai G. Netea^b^, Charles A. Dinarello^a,b^, Mark S. Gresnigt^a,d,#^

**Running title:** AAT impacts *Candida albicans* pathogenicity

**Affiliations:**

*^a^ Department of Medicine, University of Colorado Denver, Aurora*

*^b^ Department of Internal Medicine, Radboud University Medical Center and Radboud Center for Infectious diseases (RCI)*

*^c^ Radboud Institute for Molecular Life Sciences (RIMLS), Radboud University Nijmegen Medical Center (Radboudumc), Nijmegen, The Netherlands.*

*^d^ Junior Research Group Adaptive Pathogenicity Strategies, Leibniz Institute for Natural Product Research and Infection Biology – Hans Knöll Institute, Jena, Germany*

*^e^ Department of Microbial Pathogenicity Mechanisms, Leibniz Institute for Natural Product Research and Infection Biology – Hans Knöll Institute, Jena, Germany*

*^f^ Department of Laboratory Medicine, Laboratory of Hematology, Radboud University Medical Center, Nijmegen, The Netherlands.*

*^g^ Institute of Microbiology, Friedrich-Schiller-University, Jena, Germany*

*^h^ Institute of Clinical Chemistry and Pathobiochemistry, School of Medicine, Technical University of Munich, Munich, Germany.*

*^i^ Medizinische Klinik 1, Universitätsklinikum Erlangen, Friedrich-Alexander-Universität Erlangen-Nürnberg, Erlangen, Germany*

*** Contributed equally**

# **Corresponding author:**

Mark S. Gresnigt

[mark.gresnigt@leibniz-hki.de](mailto:mark.gresnigt@leibniz-hki.de)

Junior Research Group Adaptive Pathogenicity Strategies, Leibniz Institute for Natural Product Research and Infection Biology – Hans Knöll Institute, Adolf-Reichwein-Straße 23, 07745 Jena, Germany

ORCID: <https://orcid.org/0000-0002-9514-4634>

**Supplementary figure 1:**

(A) CFU-based killing assays of *C. albicans* in presence of indicated concentrations of bioactive or heat-inactivated AAT. Live *C. albicans* SC5314 yeasts cells (MOI 1) were incubated with hMDMs (4 × 10^4^/well), PMNs (1 × 10^5^/well) or monocytes (1 × 10^5^/well) and harvested for plating on YPD agar after 3 h (monocytes) or 24 h (Monocytes, Macrophages, and Neutrophils). Bars represent the mean CFU ± SEM. Bars are overlaid and represent the mean of n=8 donors (Monocytes 3h, 24h and Neutrophils 24h) n=4 donors (macrophages 24h). (B) Growth of *C. albicans* assessed by absorption at 600nm measurements in RPMI in the presence or absence of indicated concentrations of bioactive or heat-inactivated AAT (n=5 replicates).

**Supplementary figure 2:**

Individual donor plots of macrophage cell death quantification corresponding to figure 1C with over 24 h images of human macrophages infected with *C. albicans* MOI 1 in presence or absence of AAT 1 or 10 μg/mL and PI to visualize cell death events.

**Supplementary figure 3:**

Representative brightfield, red channel (propidium iodide staining of dead macrophages), and merged microscopy images from live-cell imaging for 2 out of 6 donors at 24 hours post infection from macrophages infected with *C. albicans* MOI 1 in presence or absence of AAT 1 - 1000 μg/mL

**Supplementary figure 4:**

Representative images of *C. albicans* grown in RPMI for 12h in the presence of increasing concentrations of AAT Scale bars equal 200 µm.
