# Supplementary figures and images for: Alpha1-antitrypsin impacts innate host–pathogen interactions with Candida albicans by stimulating fungal filamentation
Source: Virulence. 2024 Mar 22;15(1):2333367. doi: 10.1080/21505594.2024.2333367 (PMC11008552; doi:10.1080/21505594.2024.2333367)

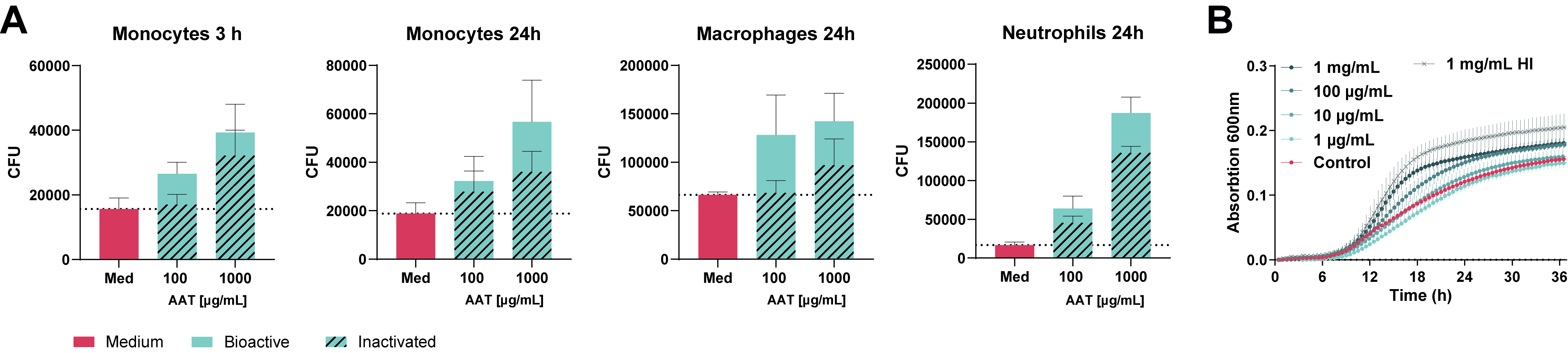

Supplement: Supplemental Material [file KVIR_A_2333367_SM4061.zip › Figure S1R1.tif]
